# Employment Status Among Brazilian Women With Estrogen Receptor‐Positive Nonmetastatic Breast Cancer

**DOI:** 10.1002/cam4.71306

**Published:** 2025-10-29

**Authors:** Daniele Assad‐Suzuki, Luciana Castro Garcia Landeiro, Danielle Laperche‐Santos, Heloisa Resende, Fernanda Cesar Moura, Sulene Cunha Sousa Oliveira, Andrea Kazumi Shimada, Renata Arakelian, Anna Luiza Zapalowski Galvão, Bruno Santos Wance de Souza, Amanda Guimarães Castro Custódio, Monalisa Ceciliana Freitas Moreira de Andrade, Yuri Cardoso Rodrigues Beckedorff Bittencourt, Maria Cristina Figueroa Magalhães, Cristiano de Pádua Souza, Carlos Eduardo Paiva, Poliana Albuquerque Signorini, Ariane Vieira Carvalho, Daniela Jessica Pereira, Laura Cereser Albaneze, Angélica Nogueira‐Rodrigues, Daniela Dornelles Rosa, Romualdo Barroso‐Sousa

**Affiliations:** ^1^ Hospital Sírio‐Libanês Brasília Brazil; ^2^ Grupo Brasileiro de Estudos Em Câncer de Mama‐ GBECAM Porto Alegre Brazil; ^3^ Oncoclínicas & co Salvador Brazil; ^4^ Oncoclínicas & co Goiânia Brazil; ^5^ Centro Universitário de Volta Redonda, UniFOA Volta Redonda Brazil; ^6^ Instituto Hospital de Base Do Distrito Federal Brasília Brazil; ^7^ Liga Norte Riograndense Contra o Câncer Natal Brazil; ^8^ Hospital Sirio Libanês São Paulo Brazil; ^9^ Hospital da Mulher e Rede Américas São Paulo Brazil; ^10^ Hospital Universitário da Universidade Federal de Juiz de Fora Juiz de Fora Brazil; ^11^ Hospital Universitário Evangélico Mackenzie Curitiba Brazil; ^12^ Barretos Cancer Hospital São Paulo Brazil; ^13^ CINPAM–Centro Integrado de Pesquisa da Amazônia Manaus Brazil; ^14^ Santa Casa de Misericórdia Belo Horizonte Brazil; ^15^ Oncocentro, Grupo Oncoclinicas Belo Horizonte Brazil; ^16^ Hospital Moinhos de Vento Porto Alegre Brazil; ^17^ Universidade de Minas Gerais Belo Horizonte Brazil; ^18^ Hospital de Clínicas de Porto Alegre Porto Alegre Brazil; ^19^ Hospital Brasília, Rede Américas Brasília Brazil

**Keywords:** breast cancer, employment, endocrine therapy, quality of life, return to work

## Abstract

**Background:**

Employment is a critical determinant of quality of life, social reintegration, and financial stability for breast cancer survivors. International studies have shown that return‐to‐work (RTW) rates vary widely, ranging from 27% to over 80% within the first 3 years postdiagnosis, and are strongly influenced by sociodemographic and systemic factors. In Brazil, however, there is a scarcity of data on employment outcomes after breast cancer, despite pronounced disparities in healthcare access between public and private systems. Understanding these dynamics is crucial to identify vulnerable groups and to inform strategies that promote equitable reintegration into the workforce.

**Methods:**

We conducted a multicenter cross‐sectional study including 454 women with nonmetastatic ER+ breast cancer who were employed at diagnosis and receiving endocrine therapy. Employment status, sociodemographic and clinical variables, and quality‐of‐life scores were collected through patient‐reported questionnaires and medical records. Univariate and multivariate generalized logit models were applied.

**Results:**

Among 774 participants, 454 (67.1%) were employed at diagnosis. Of these, 87 (19.2%) continued working during treatment, 233 (51.54%) stopped and returned, and 134 (29.29%) stopped and did not return. Compared with privately treated patients who remained employed, those treated in public hospitals had significantly higher odds of stopping work and returning (OR = 5.93) and of stopping work and not returning (OR = 2.39). Younger age (≤ 60 years) was associated with permanent work interruption (OR = 2.39). Lower education was associated with temporary interruption (OR = 3.12). Treatment duration ≥ 2 years was associated with not returning to work (OR = 2.18).

**Conclusions:**

Treatment in public hospitals, lower education, younger age, and prolonged treatment were associated with a higher risk of job loss. Addressing barriers and fostering workplace adaptations is vital for improving return‐to‐work rates among cancer survivors, as nearly one‐third do not return to work within 2 years postdiagnosis.

## Graphical abstract

A significant proportion of breast cancer survivors (nearly 1/3) stopped and did not return to work within two years postdiagnosis, with treatment in public hospitals, lower education, younger age, and prolonged treatment associated with a higher risk of job loss. Addressing barriers and promoting workplace adaptations is vital to improve return‐to‐work rates among cancer survivors.

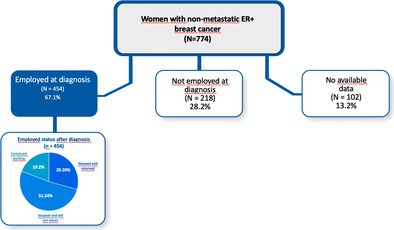

## 1 Introduction

Work is a cornerstone of economic security, social equality, and economic sustainability [1]. For cancer survivors, return to work (RTW) is a key indicator of quality of life (QOL), often representing a return to normalcy and social reintegration [2, 3]. Breast cancer remains the most common malignancy among women, with 2.3 million new cases reported globally in 2020 [4]. Advances in diagnostics and treatment have increased early detection, leading to a growing population of survivors whose employment outcomes demand closer attention [5].

In the United States (US), approximately three‐quarters of women aged 25–54 years are employed, and nearly half act as primary or co‐breadwinners [6, 7]. Breast cancer affects a significant proportion of this workforce [6]. Failure to RTW following treatment can adversely affect survivors' well‐being and financial stability [8]. RTW rates vary globally, ranging from 27% at 6 months to over 80% by 36 months postdiagnosis [8, 9]. Survivors facing older age, advanced disease, or intensive therapies often experience greater challenges with return to work, whereas those with supportive employers tend to have more favorable outcomes. Supportive employers may provide flexible working hours, gradual return‐to‐work programs, job accommodations such as reduced workload or remote work options, and protection against job loss during medical leave [8].

In Brazil, employment outcomes are influenced by unequal access to healthcare and socioeconomic disparities. Previous studies have shown that inequalities strongly influence employment outcomes after cancer in healthcare access and socioeconomic status. For instance, survivors from lower‐income backgrounds or those treated in under‐resourced public systems often experience delayed reintegration and poorer return‐to‐work outcomes compared to those treated in private facilities [10, 11]. A 2018 study in São Paulo reported a 60% return‐to‐work rate 2 years postdiagnosis among women treated in the public system. However, this single‐institution study reflects only the reality of one metropolitan area [12]. Brazil's regional socioeconomic heterogeneity and health system inequalities underscore the need for broader multicenter research to capture the diversity of patient experiences across the country. Data on employment outcomes among Brazilian breast cancer survivors are scarce, with most evidence from Europe and North America, where systemic differences limit comparability. Given the pronounced disparities between public and private care in Brazil, it is essential to investigate RTW in this context. This study evaluated employment status in women with estrogen receptor‐positive (ER+) invasive breast cancer to identify factors linked to workforce retention and the impact of adjuvant endocrine therapy (ET). We focused on women with ER+ invasive breast cancer because this subgroup represents the majority of breast cancer cases in Brazil and worldwide, and patients typically receive long‐term endocrine therapy. As a result, this population faces unique challenges in employment reintegration due to prolonged treatment duration and treatment‐related side effects, making it particularly relevant to study employment outcomes in this context. Additionally, we examined how clinical characteristics, social determinants, healthcare coverage, and QOL influence the likelihood of returning to work. By integrating sociodemographic, clinical, and QOL data across diverse healthcare settings, this study aimed to provide a comprehensive assessment of employment outcomes in Brazil.

## 2 Materials and Methods

### 2.1 Study Design and Participants

**FIGURE 1:**
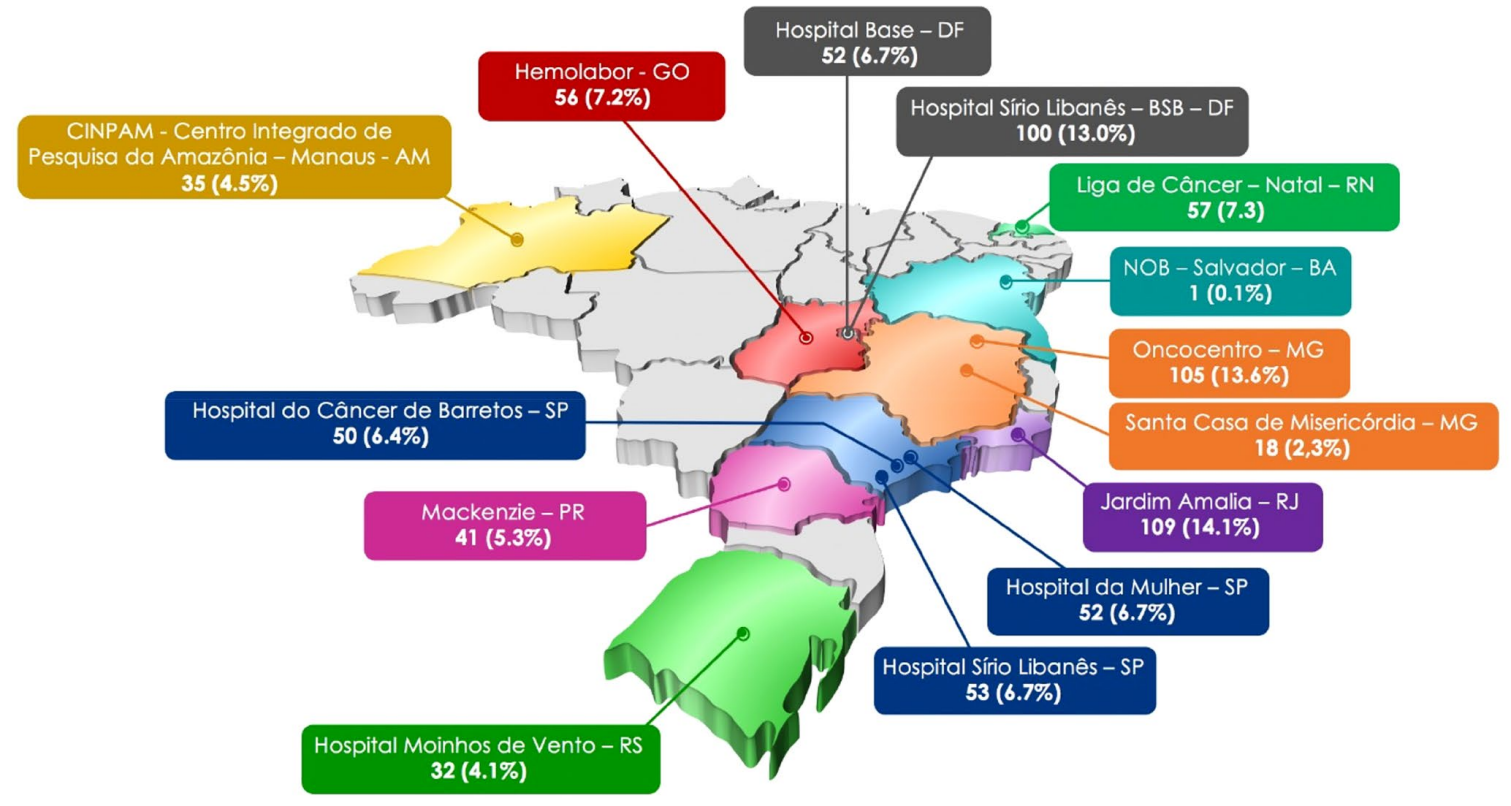
Recruitment of patients among the oncology institutions. Public institutions–Noninsured health care: Hospital do Câncer de Barretos, São Paulo (SP). Mackenzie, Paraná (PR). Hospital de Base, DF (Distrito Federal), Liga de Câncer Natal, RN (Rio Grande do Norte), Santa Casa de Misericódia, MG (Minas Gerais). Jardim Amália‐ RJ (Rio de Janeiro), Hospital da Mulher‐SP (São Paulo), CINPAM‐Manaus‐AM (Amazonas). Private institution‐insured health care: Hemolabor‐GO(Goiás), Hospital Sírio Libanês‐ Bsb (Brasília). NOB (Núcleo de Oncologia da Bahia)‐ Salvador, Oncocentro‐ MG (Minas Gerais), Hospital Sírio Libanês‐SP (São Paulo), Hospital Moinhos de Vento‐RS (Rio Grande do Sul).

This cross‐sectional study was conducted by the Grupo Brasileiro de Estudos em Câncer de Mama (GBECAM) between June 2021 and May 2023. This was a multicenter study conducted at 14 institutions across Brazil, including both public and private hospitals located in São Paulo, Rio de Janeiro, Brasília, Belo Horizonte, Porto Alegre, and Salvador (Figure 1). Patients were consecutively recruited during routine oncology follow‐up visits until the study period was completed. Eligibility was confirmed by the treating oncologist, and all participants provided written informed consent. All had received standard treatments and were on adjuvant endocrine therapy (ET) with an aromatase inhibitor (letrozole, anastrozole, exemestane) or tamoxifen, with or without ovarian suppression, for 6–120 months. Eligible participants had ECOG performance status 0–2; exclusion criteria were prior or synchronous breast cancer, other malignancies, or distant metastases.

Data were collected using a Research Electronic Data Capture (REDCap) system [13]. Medical records included demographics, medical history, histology, tumor staging and grading, receptor status (hormonal and HER2), and primary oncological treatment along with demographic variables, comorbidities, and concurrent medications. Patients were stratified by ET duration (< 2, 2–5, > 5 years), age (< 40, 40–60, > 60 years), and treatment setting (public vs. private). Employment status was categorized into three groups: (1) did not interrupt work during treatment, (2) interrupted work but returned, and (3) interrupted work and did not return.

The study received ethics approval from institutional review boards of all participating centers, with central approval at Hospital Sírio Libanês–DF (CEP. HSL 2020–58, CAAE 31294920.6.0000.5461). All participants provided written informed consent.

### 2.2 Patient‐Reported Outcomes

#### 2.2.1 Return to Work

Although one measure is now available [14], no validated patient‐reported tool existed at the time of this study to assess employment status (patient employed in a paid job before your cancer diagnosis). If yes, question about the absence from work following cancer diagnosis: already returned to work, still absent, or continued working during treatment. And about total time away from work: ≤ 6 months, 7–12 months, 13–24 months, or ≥ 24 months after cancer; therefore, a specific questionnaire was developed by our research team of oncologists, occupational health specialists, and patient representatives. The final version of the questionnaire is provided as Appendix 1.

#### 2.2.2 Health‐Related QOL

Health‐related QOL was evaluated using Brazilian Portuguese versions of the European Organisation for Research and Treatment of Cancer (EORTC) Core Quality of Life questionnaire (EORTC QLQ‐C30), and the specific module for breast cancer (QLQ‐BR23) instruments [15] (The EORTC QLQ‐BR23 was updated to the provisional QLQ‐BR45; the revised QLQ‐BR42 is available for use now [16]).

### 2.3 Statistical Analysis

This study evaluated employment status in women with estrogen receptor‐positive (ER+) invasive breast cancer to identify factors linked to workforce retention and the impact of adjuvant endocrine therapy (ET).

Baseline comparisons referred to descriptive analyses of sociodemographic, clinical, and treatment‐related characteristics across the three employment status groups. Independent variables included age, education, marital status, comorbidities, tumor stage, type of treatment, and treatment setting (public vs. private), while dependent variables were employment status categories and quality‐of‐life scores. Our primary hypothesis proposed that treatment setting (public vs. private), younger age, and lower education would be associated with increased risk of work interruption or failure to return to work.

Baseline comparisons were performed using chi‐squared or Fisher exact test for categorical variables, and Mann–Whitney test for continuous variables. Comparisons across postdiagnosis employment status groups used the chi‐squared or Fisher exact test for categorical data, and Kruskal–Wallis test for continuous data, followed by the Dwass, Steel, Critchlow‐Fligner (DSCF) test when appropriate (*p* < 0.05). Cases with missing data were not included in the respective analyses. A generalized logit model was applied to assess factors associated with employment status (reference: did not stop working), with odds ratios (OR) and 95% confidence intervals and was fitted using a stepwise selection procedure (entry/removal criteria *p* ≤ 0.05), including all demographic, clinical, treatment, and QOL variables of interest. Multicollinearity was assessed (tolerance > 0.60) and no problematic collinearity was observed. Because of the cross‐sectional design of this study, we could not establish the temporal sequence between changes in employment status and quality‐of‐life (QOL) outcomes. In line with previous RTW literature, QOL scores were analyzed as dependent variables in exploratory analyses, acknowledging that they may either reflect the impact of employment disruption or represent pre‐existing factors influencing employment outcomes. All analyses were conducted using SAS 9.4 and R (R Core Team, 2022).

## 3 Results

### 3.1 Demographics

A total of 454 patients were included in the employment status analysis. The mean age of patients who worked at diagnosis was 52 (±9.84) years (Table 1). A significant proportion (202; 60.12%) had health insurance and received treatment in private hospitals. Some 269 (59.5%) patients were living with a partner, 259 (57%) were postmenopausal, and 44 (9.7%) were older than 65 years. The cohort was ethnically diverse, with 200 (44.9%) identifying as non‐White. Disease staging revealed 147 (32.4%) patients in Stage I disease, 194 (42.7%) patients in Stage II, and 113 (24.9%) patients in Stage III. Surgical interventions included lumpectomy in 265 (58.9%) patients and axillary lymph node dissection in 142 (31.8%) patients. Prior treatment included chemotherapy in 339 (74.8%) patients and radiotherapy in 378 (83.4%) patients. Additionally, 256 (56.4%) patients had at least one comorbidity.

**TABLE 1:** Patient characteristics and treatment details related to employment status.

Patient characteristics	Employment status after cancer treatment among those working prior to cancer diagnosis (*n* = 454)			
	Did not stop working *n* (%)	Stopped work and returned *n* (%)	Stopped work and did not return *n* (%)	*p*
Employment (*n*, %)	87 (19.2)	133 (29.29%)	234 (51.54%)	
Age (mean ± SD)	53.84 ± 11.15	53.99 ± 9.91	50.70 ± 9.03	0.0116
Health insurance				< 0.0001
Yes	60 (68.97)	26 (19.55)	116 (49.57)	
No	27 (31.03)	107 (80.45)	118 (50.43)	
Menopausal status				0.6828
Premenopause	35 (40.23)	61 (45.86)	99 (42.31)	
Menopause	52 (59.77)	72 (54.14)	135 (57.69)	
Race				0.0402
White	50 (58.82)	60 (45.80)	135 (58.95)	
Non‐White	35 (41.18)	71 (54.20)	94 (41.05)	
Age				0.0039
≤ 65 years	72 (82.76)	117 (87.97)	221 (94.44)	
> 65 years	15 (17.24)	16 (12.03)	13 (5.56)	
Education level				< 0.0001
Low	10 (11.90)	63 (50.40)	26 (11.61)	
High	74 (88.10)	62 (49.60)	198 (88.39)	
Comorbidities				0.9332
Yes	37 (42.53)	57 (42.86)	104 (44.44)	
No	50 (57.47)	76 (57.14)	130 (55.56)	
Clinical stage				0.0007
I	40 (45.98)	28 (21.05)	79 (33.76)	
II	30 (34.48)	59 (44.36)	105 (44.87)	
III	17 (19.54)	46 (34.59)	50 (21.37)	
PS ECOG				0.2558
PS 0	81 (93.10)	124 (93.23)	226 (96.58)	
PS 1	6 (6.90)	9 (6.77)	8 (3.42)	
Concomitant medication				0.8923
No	43 (49.43)	64 (48.12)	109 (46.58)	
Yes	44 (50.57)	69 (51.88)	125 (53.42)	
Chemotherapy				0.0321
Yes	30 (34.48)	25 (18.80)	59 (25.32)	
No	57 (65.52)	108 (81.20)	174 (74.68)	
Endocrine therapy				0.0801
Tamoxifen	33 (37.93)	51 (38.35)	70 (30.04)	
Aromatase inhibitor	38 (43.68)	67 (50.38)	111 (47.64)	
ET + OFS	16 (18.39)	15 (11.28)	52 (22.32)	
Axillary dissection				0.016
No	66 (77.65)	78 (59.54)	161 (69.70)	
Yes	19 (22.35)	53 (40.46)	70 (30.30)	
Type of surgery				0.1632
Modified radical mastectomy	25 (29.07)	53 (40.15)	76 (32.76)	
Adenomastectomy	8 (9.30)	4 (3.03)	19 (8.19)	
Lumpectomy	53 (61.63)	75 (56.82)	137 (59.05)	
Household				0.3148
Living alone	29 (33.33)	55 (41.67)	99 (42.49)	
Living with family/Married	58 (66.67)	77 (58.33)	134 (57.51)	
Tumoral grade				0.2344
Grade 1	20 (28.17)	15 (14.42)	40 (18.69)	
Grade 2	39 (54.93)	64 (61.54)	127 (59.35)	
Grade 3	12 (16.90)	25 (24.04)	47 (21.96)	
Duration of ET				0.1836
< 2 years	36 (41.38)	46 (34.59)	68 (29.18)	
2–5 years	35 (40.23)	61 (45.86)	126 (54.08)	
> 5 years	16 (18.39)	26 (19.55)	39 (16.74)	
CDK4/6 inhibitors				0.8058
No	84 (96.55)	130 (97.74)	226 (96.58)	
Yes	3 (3.45)	3 (2.26)	8 (3.42)	
Radiotherapy				0.1988
No	18 (20.69)	16 (12.03)	41 (17.60)	
Yes	69 (79.31)	117 (87.97)	192 (82.40)	
Tumor size (mean ± SD)	21.82 ± 17.19	24.80 ± 36.31	22.90 ± 21.13	0.7832
Duration of ET	2.97 ± 2.07	3.22 ± 2.25	3.30 ± 2.03	0.2359

*Note:* 102 (13%) patients did not answer the questions about return to work.

Abbreviations: CDK4/6, cyclin‐dependent kinase (CDK) 4/6: ECOG; ET, endocrine therapy; High education, high school graduate or higher; Low education, less than complete high school; *N*, number; OFS, ovarian function suppression; PS, performance status; SD, standard deviation.

### 3.2 Employment Status at Cancer Diagnosis

**FIGURE 2:**
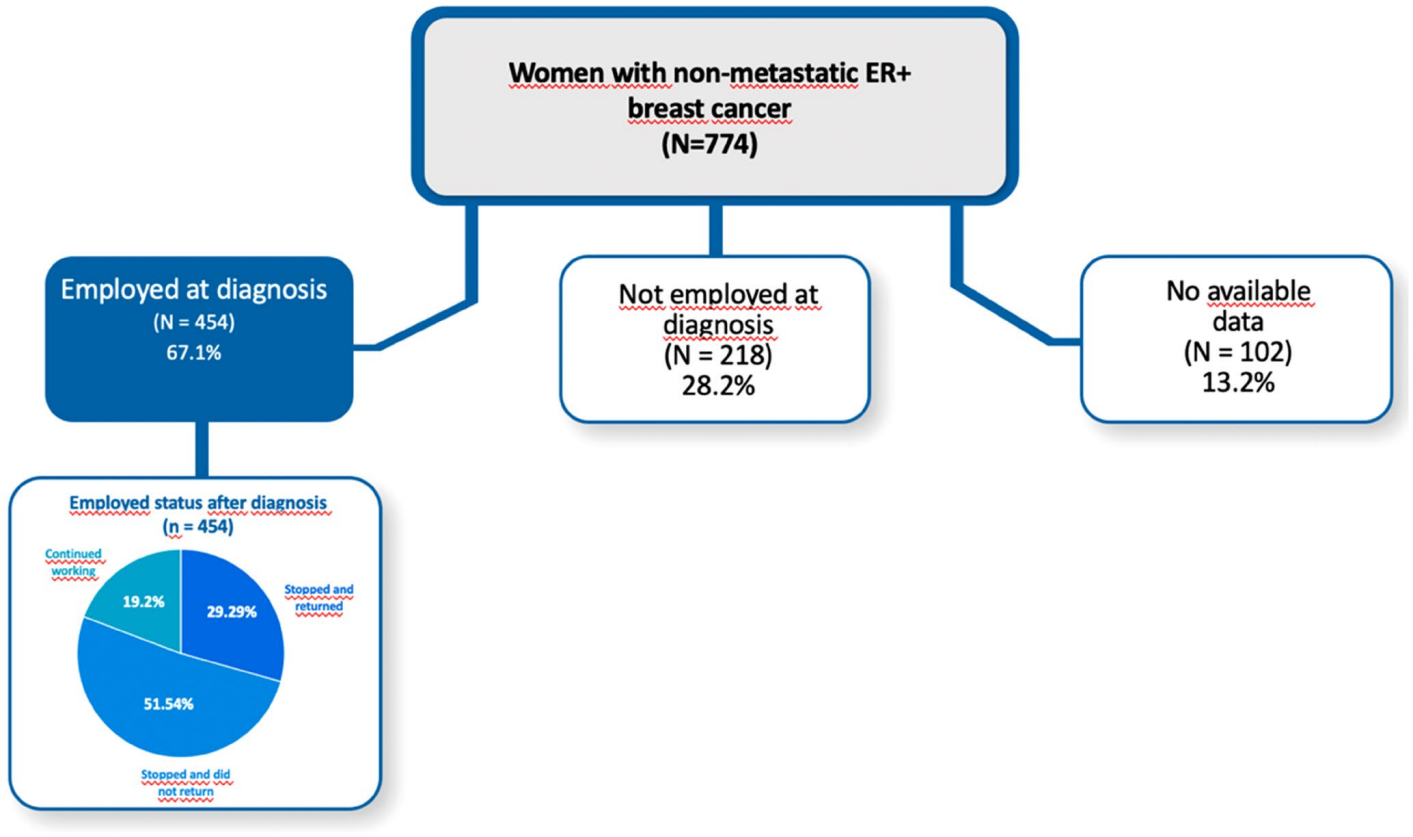
Employment status distribution among the complete cohort at diagnosis and in the employed cohort.

Of the 774 patients included, 454 (67.1%) were employed at the time of diagnosis and were therefore eligible for the RTW analyses. Patients who were not employed at diagnosis (*n* = 218) or who did not respond to RTW questions (*n* = 102) were excluded, since RTW outcomes could not be meaningfully assessed in these subgroups (Table 1). Of the 454 patients who were employed at the time of diagnosis, 51.54% did not resume work within 2 years (Figure 2). At baseline, patients who were unemployed at diagnosis were more frequently treated in public hospitals, premenopausal, and younger than 65 years, and also reported lower sexual functioning scores (*p* < 0.05). These comparisons were intended to describe baseline differences between employed and unemployed groups, rather than to indicate return‐to‐work (RTW) outcomes.

### 3.3 Univariate Analysis of Employment Status

The working rates at 6, 12, and 24 months postdiagnosis were 19.6%, 20.9%, and 23.8%, respectively, and 27.5% of the 454 employed patients did not RTW within 2 years.

In the univariate analysis (Table 2), significant associations were identified between employment status and type of hospital (*p* < 0.0001), ethnicity (*p* = 0.0402), age (*p* = 0.0039), educational level (*p* < 0.0001), clinical stage (*p* = 0.0007), axillary dissection (*p* = 0.0160), and receipt of cytotoxic chemotherapy (*p* = 0.0321). Among participants who did not stop working, the majority were treated in private hospitals (69%), had a high educational level (88%), were ≤ 65 years (83%), and were mostly diagnosed at Stage I (46%). These patients underwent axillary dissection in 22% of cases and received cytotoxic chemotherapy in 66% of cases. Among participants who were on leave and returned to work, 80% were treated in public hospitals, 50% had a low educational level, 88% were ≤ 65 years, and 35% were diagnosed at Stage III. Among those who stopped working and did not return, 50% were treated in public hospitals and 49.57% in private hospitals. Most were ≤ 65 years (94%), had a high educational level (88%), and 45% were diagnosed at Stage II.

**TABLE 2:** Univariate analysis of quality‐of‐life scores versus employment status.

Characteristics	Employment status after cancer treatment (*N* = 454)			
	Did not stop working (*n* = 87) Mean (sd)	Stopped work and did *not* return (*n* = 234) Mean (sd)	Stopped work and returned (*n* = 133) Mean (sd)	*p*
QLQ C30—Global health status	83.05 ± 20.67	78.15 ± 20.75	72.85 ± 23.79	0.002
QLQ C30—Physical functioning	89.42 ± 14.93	85.88 ± 15.32	76.44 ± 20.48	< 0.0001
QLQ C30—Role functioning	88.70 ± 22.37	85.49 ± 24.13	66.79 ± 34.30	< 0.0001
QLQ C30—Emotional functioning	69.03 ± 28.08	62.64 ± 30.95	54.67 ± 33.45	0.0053
QLQ C30—Cognitive functioning	64.94 ± 29.97	63.65 ± 31.49	54.67 ± 35.51	0.0532
QLQ C30—Social functioning	91.00 ± 20.77	87.50 ± 22.24	79.80 ± 29.92	0.0026
QLQ C30—Fatigue	17.05 ± 20.94	21.31 ± 22.62	32.15 ± 31.67	0.0023
QLQ C30—Nausea_vomiting	5.36 ± 13.33	4.81 ± 11.06	11.24 ± 18.93	0.0005
QLQ C30—Pain	19.54 ± 28.10	23.64 ± 27.23	37.50 ± 36.63	0.0002
QLQ C30—Dyspnea	5.36 ± 14.26	6.61 ± 17.94	17.54 ± 27.40	< 0.0001
QLQ C30—Sleeping disturbances	25.19 36.49	36.64 ± 39.15	44.70 ± 41.36	0.0013
QLQ C30—Appetite loss	7.36 ± 21.33	7.18 ± 19.52	12.63 ± 25.90	0.0301
QLQ C30—Constipation	19.38 ± 32.12	23.71 ± 34.09	30.05 ± 35.16	0.0235
QLQ C30—Diarrhea	3.45 ± 12.49	5.17 ± 16.18	8.08 ± 21.84	0.2383
QLQ C30—Financial difficulties	15.33 ± 28.21	15.09 ± 28.89	39.65 ± 41.42	< 0.0001
QLQ BR23—Systemic therapy side effects	18.04 ± 17.78	22.17 ± 16.45	27.02 ± 18.78	0.0001
QLQ BR23—Upset by hair loss	36.11 ± 34.16	34.65 ± 35.25	30.73 ± 35.29	0.5868
QLQ BR23—Arm symptoms	15.49 ± 20.93	18.86 ± 21.08	34.07 ± 28.98	< 0.0001
QLQ BR23—Breast symptoms	11.96 ± 17.42	15.78 ± 17.76	27.10 ± 26.33	< 0.0001
QLQ BR23—Body image	78.63 ± 25.11	75.10 ± 26.74	72.92 ± 29.17	0.4244
QLQ BR23—Future perspective	53.33 ± 35.34	47.81 ± 35.26	36.11 ± 34.96	0.0008
QLQ BR23—Sexual functioning	24.31 ± 20.66	24.41 ± 23.54	20.20 ± 22.18	0.1239
QLQ BR23—Sexual enjoyment	48.98 ± 28.95	49.49 ± 27.29	39.78 ± 26.88	0.0845

*Note:* Higher scores for global health and functioning indicate better functioning, whereas higher scores for symptoms indicate worse functioning.

Abbreviations: High education, high school graduate or higher; Low education, less than complete high school; sd, standard deviation.

A significant association was found with ethnicity (*p* = 0.0402). Non‐White participants accounted for 54% of those who were on leave and returned to work, compared to 41% of those who did not return, and 41% in the group who did not stop working.

A significant difference was also observed in age (*p* = 0.0113). Participants who did not RTW had a lower mean age (51 ± 9 years) compared to those who were on leave and returned (54 ± 10 years) and those who did not stop working (54 ± 11 years). Pairwise comparisons showed a difference between “Did not RTW” and “Returned to work” groups (*p* = 0.0157).

### 3.4 QOL and Employment Status

Significant differences were observed between employment status after treatment groups across multiple domains of the EORTC QLQ‐C30 and QLQ‐BR23 (Table 2). In the QLQ‐C30 Global Health Status, mean scores were higher among participants who did not stop working (83.05 ± 20.67) compared to those who did not RTW (78.15 ± 20.75) and those who returned to work (72.85 ± 23.79) (*p* = 0.0020). Pairwise comparisons showed a difference between the “Did not stop” and “Returned to work” groups (*p* = 0.0015).

Significant differences were also observed in the domains of physical functioning (PF) (*p* < 0.0001), role functioning (*p* < 0.0001), emotional functioning (*p* = 0.0053), and social functioning (*p* = 0.0026), with lower scores among those who returned to work. Symptom domains showed significantly higher levels of fatigue (*p* = 0.0023), pain (*p* = 0.0002), dyspnea (*p* < 0.0001), sleep disturbances (*p* = 0.0013), and appetite loss (*p* = 0.0301).

Additional differences were observed for nausea and vomiting (*p* = 0.0005) and constipation (*p* = 0.0235), both being more frequent among participants who returned to work. Financial difficulties were also greater in this group (mean score 39.65 ± 41.42), compared to those who did not stop working (15.33 ± 28.21) and those who did not RTW (15.09 ± 28.89) (*p* < 0.0001 for both comparisons).

Regarding the QLQ‐BR23 module, significant differences were identified in systemic therapy side effects (*p* = 0.0001), arm symptoms (*p* < 0.0001), breast symptoms (*p* < 0.0001), and future perspective (*p* = 0.0008). In these domains, participants who returned to work reported the highest symptom burden and the lowest functional scores. Pairwise comparisons showed statistically significant differences between this group and the others.

### 3.5 Multivariate Analysis of Employment Status

Compared with individuals who were treated in private hospitals and did not stop working, those treated in public hospitals were six times more likely to stop working and later RTW (OR = 5.93; 95% CI: 2.75–12.76; *p* < 0.0001), and two times more likely to stop working and not RTW (OR = 2.39; 95% CI: 1.29–4.40; *p* = 0.0056) (Table 3).

**TABLE 3:** Multivariate analysis of factors related to employment status among breast cancer survivors.

Characteristics	Odds ratio (Stopped work and returned vs did not stop working)		Odds ratio (Stopped working and did *not* return vs did not stop working)	
	OR (95% CI)	*p*	OR (95% CI)	*p*
Health insurance				
No	5.93 (2.75; 12.76)	< 0.0001	2.39 (1.29; 4.40)	0.0056
Yes (ref)	1	—	1	—
Age				
≤ 60	1.42 (0.66; 3.02)	0.3687	2.39 (1.25; 4.56)	0.0082
> 60 (ref)	1	—	1	—
Education level				
Low	3.12 (1.28; 7.62)	0.0125	0.59 (0.24; 1.44)	0.2448
High (ref)	1	—	1	—
Duration of ET				
< 2 years (ref)	1	—	1	—
≥ 2 years	1.57 (0.79; 3.10)	0.1939	2.18 (1.25; 3.82)	0.0062
Score PF	0.97 (0.95; 0.99)	0.0054	0.98 (0.96; 1.00)	0.1399
Score AS	1.02 (1.00; 1.03)	0.0459	1.00 (0.99; 1.02)	0.8276

*Note:* All variables listed were entered into the model regardless of statistical significance. Low education: less than complete high school. High education: high school graduate or higher.

Abbreviations: CI, confidence interval; ET, endocrine therapy; OR, odds ratio; Ref, reference category; Score AS, Score EORTC QLQ BR23—Arm Symptoms; Score PF, Score EORTC QLQ C30—Physical Functioning.

Age was also significant: Participants 60 years or younger were more likely to stop working and not RTW (OR = 2.39; 95% CI: 1.25–4.56; *p* = 0.0082), compared to those over 60 years who continued working. No age effect was observed for those who returned to work. Lower educational level was associated with stopping and later RTW (OR = 3.12; 95% CI: 1.28–7.62; *p* = 0.0125), although not with permanent work interruption. A treatment duration of 2 years or more was associated with higher odds of not returning to work (OR = 2.18; 95% CI: 1.25–3.82; *p* = 0.0062).

Higher PF scores were associated with lower odds of work interruption followed by return (OR = 0.97; 95% CI: 0.95–0.99; *p* = 0.0054), suggesting a protective effect. EORTC QLQ‐BR23 arm symptom (AS) scores showed a marginal association only for the group that returned to work (OR = 1.02; 95% CI: 1.00–1.03; *p* = 0.0459), but no effect on permanent interruption.

Multicollinearity among independent variables was assessed using variance inflation factors (VIF), and all values were below 5, indicating no concerning collinearity.

Results are presented in alignment with our primary hypothesis, which proposed that treatment setting (public vs. private), younger age, and lower education would be associated with an increased risk of work interruption or failure to return to work.

## 4 Discussion

Returning to work after a breast cancer diagnosis plays an important role in the survivor's social and economic recovery [10]. In our study, RTW rates at 6, 12, and 24 months were 19.6%, 20.9%, and 23.8%, respectively—significantly lower than the averages in international studies, which range from 40% to 89% [11] The CANTO study from France reported only 21% of patients failed to RTW after 2 years, while in our study, this proportion reached 27.5% [17]. The disparity may reflect systemic differences in healthcare access, labor protection, and rehabilitation support. Given that breast cancer is a major cause of long‐term sick leave, these low RTW rates underscore a pressing need for effective reintegration—particularly in countries like Brazil, where a high percentage of women are diagnosed during working age and economic dependency on their income is substantial [17, 18].

Approximately 70% of the Brazilian population lacks private health insurance and relies on the government‐subsidized public health system [19]. Generally, those with private health insurance have a higher socioeconomic status, indicating a correlation between health coverage and socioeconomic factors [20]. Our multivariate model showed patients treated in public hospitals were nearly six times (OR: 5.93) more likely to interrupt work and return later, and more than twice (OR: 2.39) as likely to stop working and not return, compared to those treated in private institutions who maintained employment. This association likely reflects systemic differences in care coordination and psychosocial support between public and private healthcare settings. While we cannot directly measure employer flexibility in our dataset, the treatment setting may serve as a proxy for broader differences in access to resources that can influence employment reintegration.

Educational attainment is another key factor influencing employment outcomes [21]. A low level of education was associated with stopping and later returning to work (OR = 3.12; 95% CI: 1.28–7.62; *p* = 0.0125), but not with permanent work discontinuation. This suggests that educational background may influence both the need to interrupt employment and the likelihood of returning, due to differences in job type, workplace flexibility, or access to social and occupational support systems. A systematic review by Islam et al. identified low education as a barrier to RTW for breast cancer survivors [11]. Additionally, a study among 125 employed women newly diagnosed with breast cancer at 6, 12, and 24 months found that 63% had completed at least high school [12]. This study highlighted the positive correlation between higher education and household income, which was associated with higher RTW [12]. Lower education often leads to physically demanding jobs, which increase the impact of treatment side effects—such as fatigue or pain—thus hindering RTW. Conversely, individuals with higher education are more likely to hold professional or administrative positions with greater flexibility, accommodation, and less physical strain, facilitating continued employment or earlier reintegration.

Studies indicate that adjuvant chemotherapy is associated with impaired RTW 1 year after surgery, even when controlling for therapy‐related side effects [20]. For instance, a study following 135 disease‐free breast cancer survivors reported that 5 years postdiagnosis, 57% maintained their pre‐diagnosis work status, while 22% had reduced working hours [21]. In our study, chemotherapy was associated with work absence in the univariate analysis (*p* = 0.03); however, this effect was not substantiated in the multivariate model. Hence, while chemotherapy may initially influence work disruption, its impact may be mediated by factors such as the type of hospital where patients received care and the overall burden of symptoms.

Although patients with Stages II and III breast cancer were more likely to be absent from work than those with Stage I disease in the univariate analysis, this association did not remain significant in the multivariate model. This suggests that the effect of cancer stage on employment status may be mediated by treatment intensity or institutional factors, such as access to rehabilitation and type of care. This interpretation aligns with studies indicating that women diagnosed at more advanced stages face greater challenges in returning to work [17]. The need for adjuvant therapies likely contributes to prolonged recovery and delayed workforce reintegration. A Taiwanese study involving over 23,000 patients also found that cancer stage and chemotherapy were significantly associated with work absence [20].

Ethnicity has been linked to RTW rates, as shown in a US study where Latina patients had lower RTW than non‐Latina White patients at 6 and 18 months postdiagnosis [9]. In a systematic review, non‐White and racial/ethnic minority workers were less likely to RTW following a nonoccupational injury or illness [22]. In our study, however, no association was observed between ethnicity and RTW. This discrepancy reflects differences in how ethnicity intersects with socioeconomic status. In Brazil, racial and ethnic identities are strongly associated with income level, education, and access to health care, which may act as more direct determinants of employment outcomes. As a result, when controlling for these variables, ethnicity alone may not be significant. This finding underscores the importance of addressing broader structural and socioeconomic disparities, which may have a greater impact on workforce reintegration than ethnicity.

Our results indicate that QOL significantly impacts RTW. Women who continued working had higher global health and functioning scores and fewer symptoms. Those who returned after leave had lower functioning and greater symptom burden. Notably, higher physical functioning scores were associated with a lower chance of work interruption followed by RTW (OR = 0.97; *p* = 0.0054). Arm symptoms were particularly relevant, yet despite the importance of functional outcomes, they have been underexplored. Axillary dissection was related to employment status in our study and may contribute to arm symptoms. In a prospective study in Portugal, axillary surgery was the only treatment associated with employment status [23]. Structured rehabilitation—focusing on pain management, mobility, and upper limb function—can facilitate RTW. Occupational therapy, educational interventions, ergonomic adjustments, and collaboration with employers are essential to reduce barriers. In a study of 149 patients who underwent breast cancer‐related surgery in Italy, 73.9% of patients returned to work 6 months postsurgery [21]. This study found that baseline perception of physical QOL was a predictor of RTW at follow‐up, indicating that women who viewed themselves as physically healthy were more likely to RTW after surgery. Personal evaluations and beliefs about self‐efficacy influence women's behaviors and decisions regarding RTW [21].

The relationship between age and RTW was consistent. In our study, participants who did not RTW were younger than those who returned or never stopped working. Individuals 60 years or younger had over twice the odds of not returning to work. This finding contrasts with the literature, which typically indicates that older survivors are less likely to RTW, often due to comorbidities, proximity to retirement, and reduced financial pressure [24, 25]. However, our results may reflect context‐specific dynamics in Brazil. Younger women may be engaged in informal or less secure employment, lacking labor protections that facilitate RTW. They may also experience greater distress or disruption in career development, which hinders reintegration. Additionally, younger survivors may be more likely to prioritize full recovery or face structural barriers without support for re‐entry. Contrary to prevailing literature, our study found younger breast cancer survivors were less likely to RTW, highlighting the influence of informal employment, job insecurity, and structural barriers in the Brazilian context [26]. This suggests that age is not a sole determinant of RTW, but interacts with employment type, job security, and access to support systems.

Treatment duration was significantly associated with work discontinuation. Patients who underwent treatment for 2 years or more had over twice the odds of unemployment (OR = 2.18; 95% CI: 1.25–3.82; *p* = 0.0062). A study that evaluated fluctuations in employment status with QOL 2, 3, and 5 years after cancer diagnosis revealed a major decline in employment after the first 2 years of diagnosis [27]. This highlights the cumulative impact of prolonged therapy. Extended treatment often reflects more aggressive disease or complex therapeutic regimens, which may lead to greater physical and emotional burdens, prolonged symptomatology, and extended absence from the workplace. Additionally, longer absence from the labor market may result in loss of job positions and diminished confidence in work ability. This reinforces the importance of early planning for work reintegration, especially for patients expected to undergo prolonged therapies.

This study had some limitations, including the small number of participants under 40 years, which may affect the assessment of age‐related factors. Additionally, the absence of patients from northern Brazil limits the generalizability of findings. We did not evaluate specific job types or the number of dependent children, both of which could influence RTW. Furthermore, reasons for difficulties in RTW were not assessed. Also, 13% of patients did not respond to questions related to employment status posttreatment, which may have introduced selection bias and impacted the generalizability of findings. Given the cross‐sectional design, QOL scores were analyzed as dependent variables, in line with prior RTW studies, acknowledging that the temporal sequence cannot be fully determined and causal inference cannot be made.

In summary, our study confirms that systemic and sociodemographic factors such as treatment setting, age, education, and treatment duration are key predictors of employment outcomes among Brazilian breast cancer survivors. These findings align with international evidence and underscore the need for context‐specific strategies to support return to work. While systemic and patient‐centered variables such as psychosocial support and employer accommodations have been highlighted in other studies, these were not directly assessed in our cohort. Future prospective research should investigate these mechanisms and evaluate targeted interventions aimed at improving employment reintegration.

## 5 Conclusions

Women with breast cancer undergoing adjuvant ET face barriers to RTW. In this study, more than 1/3 of patients who were employed before their cancer diagnosis were unemployed within 2 years after diagnosis. Health insurance, young age, and better QOL were associated with higher RTW rates. Identifying barriers and adapting work to survivors' needs are crucial to increasing RTW after treatment, which benefits patients' health and QOL, and society as a whole.

## Author Contributions

**Daniele Assad‐Suzuki:** conceptualization (lead), data curation (equal), formal analysis (equal), funding acquisition (equal), investigation (equal), methodology (equal), project administration (lead), resources (equal), supervision (equal), validation (equal), visualization (equal), writing – original draft (lead), writing – review and editing (lead). **Luciana Castro Garcia Landeiro:** conceptualization (equal), formal analysis (equal), methodology (equal), writing – original draft (equal), writing – review and editing (equal). **Danielle Laperche‐Santos:** conceptualization (equal), data curation (equal), formal analysis (equal), investigation (equal), methodology (equal), writing – original draft (equal), writing – review and editing (equal). **Heloisa Resende:** data curation (equal), investigation (equal), methodology (equal), visualization (equal), writing – original draft (equal), writing – review and editing (equal). **Fernanda Cesar Moura:** data curation (equal), investigation (equal), methodology (equal), writing – original draft (equal), writing – review and editing (equal). **Sulene Cunha Sousa Oliveira:** data curation (equal), formal analysis (equal), investigation (equal), writing – original draft (equal), writing – review and editing (equal). **Andrea Kazumi Shimada:** conceptualization (equal), investigation (equal), methodology (equal), validation (equal), visualization (equal), writing – original draft (equal), writing – review and editing (equal). **Renata Arakelian:** investigation (equal), methodology (equal), writing – original draft (equal), writing – review and editing (equal). **Anna Luiza Zapalowski Galvão:** investigation (equal), writing – review and editing (equal). **Bruno Santos Wance de Souza:** investigation (equal), methodology (equal), resources (equal), software (equal), supervision (equal), validation (equal), visualization (equal). **Amanda Guimarães Castro Custódio:** conceptualization (equal), investigation (equal), methodology (equal), visualization (equal), writing – review and editing (equal). **Monalisa Ceciliana Freitas Moreira de Andrade:** investigation (equal), writing – review and editing (equal). **Yuri Cardoso Rodrigues Beckedorff Bittencourt:** investigation (equal), methodology (equal), writing – review and editing (equal). **Maria Cristina Figueroa Magalhães:** conceptualization (equal), investigation (equal), methodology (equal), writing – review and editing (equal). **Cristiano de Pádua Souza:** investigation (equal), methodology (equal), writing – review and editing (equal). **Carlos Eduardo Paiva:** investigation (equal), methodology (equal), writing – review and editing (equal). **Poliana Albuquerque Signorini:** investigation (equal), methodology (equal), writing – review and editing (equal). **Ariane Vieira Carvalho:** investigation (equal), writing – review and editing (equal). **Daniela Jessica Pereira:** investigation (equal), writing – review and editing (equal). **Laura Cereser Albaneze:** investigation (equal), writing – review and editing (equal). **Angélica Nogueira‐Rodrigues:** conceptualization (equal), data curation (equal), formal analysis (equal), investigation (equal), methodology (equal), writing – original draft (equal), writing – review and editing (equal). **Daniela Dornelles Rosa:** conceptualization (equal), investigation (equal), methodology (equal), project administration (equal), writing – review and editing (equal). **Romualdo Barroso‐Sousa:** conceptualization (equal), data curation (equal), formal analysis (equal), funding acquisition (equal), methodology (equal), project administration (equal), supervision (equal), validation (equal), writing – review and editing (equal).

## Ethics Statement

Ethical approval was obtained from the research ethics committee of each participating site, and Institutional Review Board approval was obtained from the coordinating center Hospital Sírio Libanês (DF) (CEP N° HSL 2020–58, CAAE 31294920.6.0000.5461, Plataforma Brasil: 4.020.472).

## Consent

All participants signed a written informed consent form before inclusion in the study. The authors affirm that human research participants provided informed consent for publication.

## Conflicts of Interest

D.A.‐S. received speaker bureau fees from AstraZeneca, Daichi Sankyo, Lilly, Novartis, Merck, and Roche. She has also served as a consultant/advisor to AstraZeneca, Novartis, and Daichi Sankyo. She received support from AstraZeneca, Roche, and Daichi Sankyo for attending the medical conferences. The authors received institutional research funding from Daichi Sankyo and Lilly and Company. L.C.G.L. has served as a consultant/advisor to Daichi Sankyo and AstraZeneca. She received support from Libbs Farmacêutica, Pfizer, and Merck for attending the medical conferences. D.L.‐S. reported receiving speaker bureau fees from AstraZeneca, Daichi Sankyo, Lilly, Merck, and Roche. She has also served as a consultant/advisor for AstraZeneca and Roche and has received support for attending medical conferences from AstraZeneca, Roche, Lilly, Daichi Sankyo, Amgen, Novartis, and Merck. F.C.M. reported receiving speaker bureau fees from AstraZeneca, Daiichi Sankyo, Novartis, Merck, and Roche. B.S.W.S. is the CEO of Oncoliv startup. S.C.S.O. reported receiving speaker bureau fees from AstraZeneca, Daiichi Sankyo, GSK, Novartis, Astellas, and Janssen. She has also served as a consultant/advisor to AstraZeneca and Daiichi Sankyo. She received support from AstraZeneca for attending the medical conferences. A.K.S. has reported receiving speaker bureau fees from AstraZeneca, Daichi Sankyo, Lilly, Novartis, Pfizer, MSD, Janssen, Adium, Pint Pharma, Gilead, and Roche. She has also served as a consultant/advisor to AstraZeneca, Daichi Sankyo, and Pfizer. She received support from Pfizer, Roche, and Daichi Sankyo for attending medical conferences. R.A. reported receiving speaker bureau fees from AstraZeneca, Daiichi Sankyo, Lilly, Novartis, Merck, and Gilead. She has also served as a consultant/advisor for AstraZeneca and Daiichi Sankyo and has received support for attending medical conferences from Novartis and AstraZeneca. Y.C.R.B.B. reported receiving speaker bureau fees from AstraZeneca, Daiichi Sankyo, Novartis, Pfizer, Merck, Adium, and Pint Pharma. M.C.F.M. reported receiving bureau fees from Novartis, Lilly, Bristol‐Myers Squibb, Knight, Merck, Daiichi Sankyo, AstraZeneca, Pint Pharma, GSK, Roche, Gilead, and Adium. She has also served as a consultant/advisor to Novartis, AstraZeneca, Adium, MSD, and Lilly. She has participated as an investigator/subinvestigator in studies sponsored by the pharmaceutical industry. She received support for attending medical conferences from AstraZeneca, Daiichi Sankyo, and Novartis. She is the Vice President of INTES (Instituto de Inovação e Ensino em Saúde) [Institute for Education and Innovation in Health]. P.A.S. received speaker fees from AstraZeneca, Lilly, Pfizer, Novartis, Johnson and Johnson, Takeda, and Adium. A.N.‐R. Received personal honoraria for advisory boards and lectures from AbbVie, AstraZeneca, Daiichi Sankyo, Eisai, Gilead, GSK, Immunogen, Merck, Novartis, Pfizer, Roche; President elect for the Brazilian Society of Medical Oncology (uncompensated); Chair LACOG (uncompensated); Director of Strategic Planning Brazilian Group of Gynecologic Cancer (uncompensated); and Director International Affairs Brazilian Group of Studies in Breast Cancer (uncompensated). D.D.R. reported served as a consultant for Roche, Novartis, AstraZeneca, Lilly, GSK, Sanofi, Libbs, Eisai, Pfizer, Dr. Reddy's, United Medical, and Daiichi Sankyo. She received research funding from Amgen, Roche, GSK, and L'Oréal. She provided expert testimony for Roche, Novartis, Pfizer, AstraZeneca, Lilly, Teva, and Gilead. R.B.‐S. reported receiving speaker bureau fees from Agilant, AstraZeneca, Daichi Sankyo, Lilly, Pfizer, Novartis, Merck, and Roche. He also served as a consultant/advisor for AstraZeneca, Lilly, Libbs, Roche, and Merck and received support for attending medical conferences from AstraZeneca, Roche, Lilly, Daichi Sankyo, and Merck. The authors received institutional research funding from AstraZeneca and Daichi Sankyo. He holds stock in RD Medicine—Educação Médica LTDA. H.R., A.L.Z.G., A.G.C.C., M.C.F.M.A., C.P.S., C.E.P., A.V.C., D.J.P., L.C.A. declares no conflicts of interest.

## Data Availability

The data that support the findings of this study are available from the corresponding author upon reasonable request.

## Appendix 1

Patients were asked to complete a questionnaire developed by our research team regarding their employment status prior to their cancer diagnosis. If the patient was employed before the diagnosis, the following additional questions were presented.

Were you employed in a paid job prior to your cancer diagnosis?

- No
- Yes

If you answered “Yes,” please answer the following questions:

1. Were you absent from work following your cancer diagnosis?
  - Yes, and I have already returned to work.
  - Yes, and I am still absent.
  - No, I continued working during treatment.
2. Total time away from work:
  - ≤ 6 months
  - 7–12 months
  - 13–24 months
  - ≥ 24 months

## References

[1] M. A.‐O. Gjellestad , K. A.‐O. X. Haraldstad , H. Enehaug , and M. A.‐O. Helmersen , “Women's Health and Working Life: A Scoping Review,” International Journal of Environmental Research and Public Health 20 (2023): 1080, 10.3390/ijerph20021080. 36673834 PMC9859470

[2] M. Lavdaniti , D. A. Owens , P. Liamopoulou , et al., “Factors Influencing Quality of Life in Breast Cancer Patients Six Months After the Completion of Chemotherapy,” Diseases 7 (2019): 26, 10.3390/diseases7010026. 30813488 PMC6473656

[3] J. F. Steiner , T. A. Cavender , D. S. Main , and C. J. Bradley , “Assessing the Impact of Cancer on Work Outcomes,” Cancer 101, no. 8 (2004): 1703–1711. 15386303 10.1002/cncr.20564

[4] H. A.‐O. Sung , J. Ferlay , R. A.‐O. Siegel , et al., “Global Cancer Statistics 2020: GLOBOCAN Estimates of Incidence and Mortality Worldwide for 36 Cancers in 185 Countries,” CA: A Cancer Journal for Clinicians 71 (2021): 209–249. 33538338 10.3322/caac.21660

[5] M. Arnold , E. Morgan , H. Rumgay , et al., “Current and Future Burden of Breast Cancer: Global Statistics for 2020 and 2040,” Breast 66 (2022): 15–23. 36084384 10.1016/j.breast.2022.08.010 PMC9465273

[6] E. J. Cathcart‐Rake , K. J. Ruddy , A. Bleyer , and R. H. Johnson , “Breast Cancer in Adolescent and Young Adult Women Under the Age of 40 Years,” JCO Oncology Practice 17 (2021): 305–313. 33449828 10.1200/OP.20.00793

[7] “Fact Sheet: The State of Women in the Labor Market in 2023 [20/04/2024],” (2023), https://www.americanprogress.org/about‐us/.

[8] S. Q. Fantoni , C. Peugniez , A. Duhamel , J. Skrzypczak , P. Frimat , and A. Leroyer , “Factors Related to Return to Work by Women With Breast Cancer in Northern France,” Journal of Occupational Rehabilitation 20, no. 1 (2010): 49–58, 10.1007/s10926-009-9215-y. 19902340

[9] V. S. Blinder , S. Patil , A. Thind , et al., “Return to Work in Low‐Income Latina and Non‐Latina White Breast Cancer Survivors: A 3‐Year Longitudinal Study,” Cancer 15, no. 6 (2012): 1664–1674, 10.1002/cncr.26478. PMC3263326 22009703

[10] A. Mehnert , “Employment and Work‐Related Issues in Cancer Survivors,” Critical Reviews in Oncology/Hematology 77 (2011): 109–130. 20117019 10.1016/j.critrevonc.2010.01.004

[11] T. Islam , M. Dahlui , H. A. Majid , et al., “Factors Associated With Return to Work of Breast Cancer Survivors: A Systematic Review,” BMC Public Health 14 (2014): S8. 10.1186/1471-2458-14-S3-S8 PMC4251139 25437351

[12] L. C. G. Landeiro , D. M. Gagliato , A. B. Fêde , et al., “Return to Work After Breast Cancer Diagnosis: An Observational Prospective Study in Brazil,” Cancer 124, no. 24 (2018): 4700–4710. 30329152 10.1002/cncr.31735

[13] P. A. Harris , R. Taylor , R. Thielke , J. Payne , N. Gonzalez , and J. G. Conde , “Research Electronic Data Capture (REDCap)–A Metadata‐Driven Methodology and Workflow Process for Providing Translational Research Informatics Support,” Journal of Biomedical Informatics 42 (2009): 377–381. 18929686 10.1016/j.jbi.2008.08.010 PMC2700030

[14] C. Maheu , M. Singh , W. L. Tock , et al., “The Cancer and Work Scale (CAWSE): Assessing Return to Work Likelihood and Employment Sustainability After Cancer,” Current Oncology 32, no. 3 (2025): 166, 10.3390/curroncol32030166. 40136370 PMC11940880

[15] C. E. Paiva , E. C. Carneseca , E. M. Barroso , et al., “Further Evaluation of the EORTC QLQ‐C30 Psychometric Properties in a Large Brazilian Cancer Patient Cohort as a Function of Their Educational Status,” Supportive Care in Cancer 22, no. 8 (2014): 2151–2160, 10.1007/s00520-014-2206-3. 24652051

[16] V. Bjelic‐Radisic , F. Cardoso , J. Weis , et al., “An International Phase IV Field Study–Psychometric Properties of the Updated Module on Assessing Quality of Life of Patients With Breast Cancer EORTC QLQ‐BR42,” Breast 80 (2025): 103890, 10.1016/j.breast.2025.103890. 39947087 PMC11867226

[17] E. Caumette , I. Vaz‐Luis , S. Pinto , et al., “The Challenge of Return to Work After Breast Cancer: The Role of Family Situation, CANTO Cohort,” Current Oncology 28 (2021): 3866–3875. 34677248 10.3390/curroncol28050330 PMC8534983

[18] “Ans. General Data Beneficiaries of Private Health Plans, by Assistance Coverage (Brazil: 2012–2022),” (2023).

[19] A. Roman , “A Closer Look Into Brazil's Healthcare System: What Can We Learn?,” Cureus 15 (2023): e38390. 37265925 10.7759/cureus.38390 PMC10231901

[20] M. E. Schmidt , S. Scherer , J. Wiskemann , and K. Steindorf , “Return to Work After Breast Cancer: The Role of Treatment‐Related Side Effects and Potential Impact on Quality of Life,” European Journal of Cancer Care 28 (2019): e13051. 31033073 10.1111/ecc.13051

[21] E. Fiabane , P. Dordoni , C. Perrone , A. Bernardo , F. Corsi , and P. Gabanelli , “What Really Matters for Returning to Work After Breast Cancer? A 6‐Month Exploratory Study,” Women & Health 64, no. 4 (2024): 298–307. 38499393 10.1080/03630242.2024.2324312

[22] A. Jetha , L. Navaratnerajah , F. V. Shahidi , et al., “Racial and Ethnic Inequities in the Return‐To‐Work of Workers Experiencing Injury or Illness: A Systematic Review,” Journal of Occupational Rehabilitation 33, no. 3 (2023): 432–449, 10.1007/s10926-023-10119-1. 37294368 PMC10495511

[23] I. Monteiro , S. Morais , A. R. Costa , et al., “Changes in Employment Status up to 5 Years After Breast Cancer Diagnosis: A Prospective Cohort Study,” Breast 48 (2019): 38–44, 10.1016/j.breast.2019.07.007. 31493581

[24] K. A.‐O. Lamore , T. Dubois , U. Rothe , et al., “Return to Work Interventions for Cancer Survivors: A Systematic Review and a Methodological Critique,” International Journal of Environmental Research and Public Health 16 (2019): 1343, 10.3390/ijerph16081343. 31014004 PMC6518012

[25] A. G. E. M. de Boer , S. Torp , A. Popa , et al., “Long‐Term Work Retention After Treatment for Cancer: A Systematic Review and Meta‐Analysis,” Journal of Cancer Survivorship 14 (2020): 135–150. 32162193 10.1007/s11764-020-00862-2 PMC7182621

[26] S. M. Rosenberg , I. Vaz‐Luis , J. Gong , et al., “Employment Trends in Young Women Following a Breast Cancer Diagnosis,” Breast Cancer Research and Treatment 177, no. 1 (2019): 207–214, 10.1007/s10549-019-05293-x. 31147983 PMC7265819

[27] S. J. Tamminga , L. P. Jansen , M. H. W. Frings‐Dresen , and A. G. E. M. de Boer , “Long‐Term Employment Status and Quality of Life After Cancer: A Longitudinal Prospective Cohort Study From Diagnosis up to and Including 5 Years Post Diagnosis,” Work 66, no. 4 (2020): 901–907, 10.3233/WOR-203234. 32925145 PMC7683081

